# Cost-effectiveness of an autoantibody test (*Early*CDT-Lung) as an aid to early diagnosis of lung cancer in patients with incidentally detected pulmonary nodules

**DOI:** 10.1371/journal.pone.0197826

**Published:** 2018-05-22

**Authors:** John Edelsberg, Derek Weycker, Mark Atwood, Geoffrey Hamilton-Fairley, James R. Jett

**Affiliations:** 1 Policy Analysis Inc. (PAI), Brookline, MA, United States of America; 2 Oncimmune Ltd, Nottingham, United Kingdom; 3 Oncimmune LLC, Napa, CA, United States of America; Dartmouth College Geisel School of Medicine, UNITED STATES

## Abstract

**Objective:**

Patients who have incidentally detected pulmonary nodules and an estimated intermediate risk (5–60%) of lung cancer frequently are followed via computed tomography (CT) surveillance to detect nodule growth, despite guidelines for a more aggressive diagnostic strategy. We examined the cost-effectiveness of an autoantibody test (AABT)—Early Cancer Detection Test-Lung (EarlyCDT-Lung^TM^)—as an aid to early diagnosis of lung cancer among such patients.

**Methods:**

We developed a decision-analytic model to evaluate use of the AABT versus CT surveillance alone. In the model, patients with a positive AABT—because they are at substantially enhanced risk of lung cancer—are assumed to go directly to biopsy, resulting in diagnosis of lung cancer in earlier stages than under current guidelines (a beneficial stage shift). Patients with a negative AABT, and those scheduled for CT surveillance alone, are assumed to have periodic CT screenings to detect rapid growth and thus to have their lung cancers diagnosed—on average—at more advanced stages.

**Results:**

Among 1,000 patients who have incidentally detected nodules 8–30 mm, have an intermediate-risk of lung cancer, and are evaluated by CT surveillance alone, 95 (9.5%) are assumed to have lung cancer (local, 73.6%; regional, 22.0%; distant, 4.4%). With use of the AABT set at a sensitivity/specificity of 41%/93% (stage shift = 10.8%), although expected costs would be higher by $949,442 ($949 per person), life years would be higher by 53 (0.05 per person), resulting in a cost per life-year gained of $18,029 and a cost per quality-adjusted life year (QALY) gained of $24,330. With use of the AABT set at a sensitivity/specificity of 28%/98% (stage shift = 7.4%), corresponding cost-effectiveness ratios would be $18,454 and $24,833.

**Conclusions:**

Under our base-case assumptions, and reasonable variations thereof, using AABT as an aid in the early diagnosis of lung cancer in patients with incidentally detected pulmonary nodules who are estimated to be at intermediate risk of lung cancer and are scheduled for CT surveillance alone is likely to be a cost-effective use of healthcare resources.

## Introduction

The widespread use of chest computerized tomography (CT) to investigate intrathoracic diseases commonly results in the incidental detection of pulmonary nodules. Gould and colleagues estimated that 1.5 million nodules 4–30 mm in diameter are detected annually in the US, and that 55% of all nodules are >8 mm [1]. Because the probability of malignancy is directly proportional to nodule diameter, the prevalence of lung cancer is high among incidentally detected solid pulmonary nodules in this size range.

In the evaluation of such incidentally detected nodules, the goal is detection of malignancy before progression to higher stages (with their associated poor prognosis), while minimizing the costs and harms resulting from the work-up of benign nodules. The best strategy for achieving this goal is, however, currently unclear. Recent guidelines for nodules >8 mm from the Fleischner Society are quite general, suggesting CT, positron emission tomography (PET) coupled with CT (PET-CT), or tissue sampling at 3 months, “depending on size, morphology, comorbidity, and other factors,” with additional diagnostic testing at 9 and 24 months for those patients with prior negative results [2]. Guidelines from the American College of Chest Physicians (ACCP) for nodules in this size range are more detailed [3]. For solid nodules >8 mm with a 5–60% predicted probability of malignancy (as determined intuitively or via a risk equation), PET or PET-CT is recommended, with immediate biopsy for patients with a positive scan (since this result greatly increases the probability that the nodule is malignant) and, for patients with negative scans, periodic CT follow-up over 24 months to detect rapid growth.

Several studies have documented physicians’ failure to follow guidelines for the evaluation of incidentally detected pulmonary nodules [4–7]. Our study focuses on the large group of patients with intermediate-risk nodules who, despite recommendations for PET-CT, are followed by CT surveillance alone. To the best of our knowledge, only one study of guideline compliance provides sufficient data concerning use of diagnostic modalities to enable estimation of both the extent of the problem and the risk of cancer among patients of interest. In this study, Tanner and colleagues analyzed 337 patients who were referred to pulmonologists and thoracic surgeons for evaluation of pulmonary nodules 8–30 mm in diameter; 74 of the 174 patients (42.5%) who were estimated by these physicians to have an intermediate risk (5–60%) of malignancy were initially followed by CT alone, and of these, 9.5% had lung cancer [6].

Concerns about the accuracy of PET-CT may be an important reason for its omission. In a study population from a large CT screening trial, the sensitivity of PET/CT, although near-perfect (98%) for nodules ≥15 mm in diameter, was documented to be substantially worse (65%) for smaller nodules [8]. Moreover, the specificity of PET-CT, especially in regions of the country where infectious lung diseases are endemic, is less than ideal. In a meta-analysis of studies of PET and PET-CT for evaluation of pulmonary nodules, the specificity was 77% in non-endemic regions and 61% in endemic regions [9]. Thus, if PET-CT is performed among intermediate-risk patients, most of whom are at the low end of the risk range, a large majority of positive tests will be false-positives and biopsies will be negative for cancer.

In our study, we examine whether initial administration of an autoantibody test (AABT) to intermediate-risk patients, in lieu of CT surveillance alone, could improve outcomes in a cost-effective manner. An autoantibody test (AABT) for lung cancer—Early Cancer Detection Test-Lung (EarlyCDT-Lung^TM^)—has been validated and made available for use in the US via the Clinical Laboratory Improvement Amendments (CLIA) [10–17]. The AABT comprises a panel of seven tumor-related antigens that have been found to be present primarily in lung cancer, but occasionally in other cancers, several years before clinical detection [10–11, 14, 18]. The AABT consistently identifies lung cancer with 92% accuracy, and has a sensitivity of 41% for all stages/types of lung cancer and a specificity of 93% for all cohorts, although the criteria chosen for test positivity may be varied to yield alternative combinations of sensitivity and specificity (e.g., 28%/98%) [12,16]. The AABT has been both technically and clinically validated in seven independent validation cohorts, and its performance characteristics have been further validated in the commercial setting [10–11, 13]. Because the AABT is less expensive than PET-CT, and because a positive test confers greatly increased probability of a nodule being malignant (S1 Appendix), its administration to intermediate-risk patients who would otherwise undergo CT surveillance alone may yield additional life years at a reasonable cost (i.e., may be cost-effective).

## Methods

### Model description

We used a decision-analytic model to evaluate the cost-effectiveness of the AABT as an aid to the early diagnosis of lung cancer. Patients in the model population are assumed to have incidentally detected nodules of diameter 8 to 30 mm (“8–30 mm”) and an estimated 5–60% risk of lung cancer. Two alternative strategies for nodule evaluation are compared—one with use of the AABT followed by biopsy if AABT-positive and CT surveillance if AABT-negative, and one without use of the AABT (i.e., CT surveillance alone), but otherwise similar in all respects.

In the first strategy, all patients receive the AABT at model entry and those with a positive AABT (a small proportion of all model patients), being at greatly increased risk of having lung cancer, go directly to diagnostic follow-up involving biopsy of the nodule via fiberoptic bronchoscopy (FOB), CT-guided transthoracic needle aspiration (CT-TTNA), or wedge resection during video-assisted thoracic surgery (VATS). Patients with a negative AABT—the great majority of patients tested—receive up to three CT screenings at the midpoint of screening intervals recommended in current ACCP guidelines (i.e., CT at 4 months, 10 months, and 21 months, as needed, i.e., until volume doubling is detected if the nodule is malignant, or until three negative (no volume doubling) CT scans have been performed, if the nodule is benign. For purposes of analysis, all patients are classified as: true-positive (i.e., malignant nodule/ positive AABT); false-positive (benign nodule/ positive AABT); true-negative (benign nodule/negative AABT); and false negative (malignant nodule/negative AABT). In the second strategy, all patients receive the same schedule of CT surveillance as do the AABT negative patients.

All malignant nodules are assumed to be diagnosed at the time of biopsy. If not diagnosed at the time of model entry (i.e., by biopsy following a positive AABT), malignant nodules are assumed to increase in size and to progress (i.e., from local to regional, from regional to distant) during the 24-month follow-up period, and are assumed to be diagnosed soon after the first CT following volume doubling/progression. Although the total number of lung cancers diagnosed over the 24-month period will be the same with both strategies, true-positive patients in the AABT strategy will be diagnosed earlier than would be the case in the comparison strategy and thus have—on average—a beneficial “stage shift,” i.e., earlier stage disease at the time of diagnosis. AABT negative patients with lung cancer (i.e., false-negatives) follow the CT surveillance strategy and thus do not benefit from a corresponding “stage shift.” Patients with benign nodules that are positive for malignancy on AABT undergo biopsy that is assumed to rule out the presence of lung cancer.

For each strategy, the model projects life-years (unadjusted and quality-adjusted) for all patients as well as expected costs including AABT, CT, biopsy (i.e., diagnostic follow-up), and treatment, as appropriate. Cost-effectiveness is calculated in terms of cost per life-year gained and cost per QALY gained, respectively.

### Model estimation

Model parameter values were estimated using published data, where available, and expert assumptions, where needed (Tables 1–2). Methods employed to estimate key parameters—including risk of lung cancer, sensitivity/specificity of AABT, stage shift, health-state utilities, and healthcare costs—are summarized below. Additional details are provided in the S1 Appendix.

**Table 1:** Population, disease, and strategy characteristics for patients who have incidentally detected pulmonary nodules, are at intermediate risk, and were scheduled for CT surveillance alone.

Model Parameter	CT Surveillance	AABT	Source
Population Characteristics			
Age (years), mean	65.3	65.3	Tanner 2017, Swensen 1997
Female, %	47.1%	47.1%	
Smoking Status (Current/Former), %	76.5%	76.5%	
Disease Characteristics			
Prevalence of Lung Cancer, %	9.5%	9.5%	Swensen 1997
NSCLC / SCLC, %	96% / 4%	96% / 4%	
Strategy Characteristics			
Sensitivity	—	41.0% / 28.0%*	Healey 2017
Specificity	—	93.0% / 98.0%*	Healey 2017
Stage Shift (vs Serial CT) †, %	—	10.8% / 7.4%	Steele 1973, Gould 2003
Distribution of Malignant Nodules, %			
Local	73.6%	84.4% / 81.0%	Steele 1973, Gould 2003
Regional	22.0%	13.0% / 15.8%	
Distant	4.4%	2.6% / 3.2%	
Overdiagnosis Bias‡, %	18.0%	18.0%	Patz 2014

AABT, autoantibody test; NSCLC, non-small-cell lung cancer; SCLC, small-cell lung cancer

*41%/93%, 28%/98%: two alternative sets of values for sensitivity/specificity of AABT only

†Based on strategy sensitivity as well as tumor doubling time (basecase = 5.3 months) and probability of disease progression during two-year follow-up (basecase = 55.3%)

‡NSCLC only

**Table 2:** Health state utilities and costs for patients who have incidentally detected pulmonary nodules, are at intermediate risk, and were scheduled for CT surveillance alone.

Model Parameter	Value	Source
Health State Utilities		
Age, years		
50–54	0.90	Fryback 1993
55–64	0.87	
65–74	0.83	
≥75	0.79	
Lung Cancer		
NSCLC		
Local	0.71	Black 2014
Regional (ie, Stage 2/3)	0.67 / 0.65	
Distant	0.62	
SCLC	0.62	Black 2014
Costs*		
AABT	$575	OncImmune
CT	$245	Black 2014
Diagnostic Follow-up	$5,415	RBRVS 2016, HCUP-NIS 2014, David 2012, Weiner 2011, Wang Memoli 2012
*Bronchoscopy (10% of patients)*	*$1*,*553*	
*CT-TTNA (60% of patients*, *and includes cost of complications)*	*$901*	
*Wedge Resection (30% of patients)*	*$15*,*730*	
Lung Cancer Treatment	$36,724	Black 2014

ABT, autoantibody test; CT, computed tomography; NSCLC, non-small-cell lung cancer; RBRVS, Resource-Based Relative Value Scale; SCLC, small-cell lung cancer

*Costs expressed in 2016 US dollars

### Prevalence of lung cancer and population characteristics

In the aforementioned Tanner study, the prevalence of malignancy in the population similar to our model population (i.e., patients with incidentally detected nodules 8–30 mm and an estimated 5–60% risk of lung cancer) was 23.6%.^6^ Although the prevalence of lung cancer among the 42.5% of patients scheduled for CT surveillance is not explicitly stated in the Tanner study, it can be readily calculated from reported data to be 9.5% (see the S1 Appendix for details of this calculation). (That the prevalence of cancer in the CT surveillance subgroup was substantially lower than the prevalence in the intermediate-risk group as a whole reflects physician skill in assigning the lowest risk patients to the most conservative follow-up strategy.)

For purposes of estimating life-expectancy, the model population was assumed to have the characteristics of the Tanner intermediate-risk population: 65.3 years of age; 47.1% female; and 76.5% current/former smokers. Because the Tanner study did not specify the percentages of lung cancer cases that were non-small cell and small-cell (NSCLC and SCLC), and because our life expectancy estimates varied somewhat for these two categories of lung cancer, we assumed that these percentages (NSCLC: 96%; SCLC: 4%) were identical to those found in a prior study of incidentally detected pulmonary nodules [19].

### Sensitivity and specificity

For the AABT, two alternative sets of estimates for sensitivity and specificity were considered in base-case analyses based on published literature: 41%/93% and 28%/98%, respectively [16].

### Stage of malignant nodules at detection

Published data concerning the stage distribution of incidentally detected lung cancers are not available. We assumed that almost all patients receiving CT surveillance had nodules at the low end of the size range (in the Tanner study, mean nodule diameter in the intermediate-risk group was 14.2 mm) due to the known lack of sensitivity of PET-CT for small lung cancers, and thus that all cancers were initially in local stages.

### Growth/Progression of malignant nodules

Progression of lung cancer during the 24-month follow-up period was projected based on volume doubling time (VDT) of malignant nodules and the probability of stage progression given volume doubling [20]. Specifically, from Figure 5 in the S1 Appendix of Gould et al., data on doubling time were used to generate monthly probabilities of tumor doubling and, using a Poisson distribution, data on disease progression were employed to generate monthly probabilities of progression given tumor doubling. These probabilities were combined to generate cumulative monthly probabilities that malignant nodules had: 1) not doubled and not progressed; 2) doubled but not progressed; and 3) doubled and progressed. Malignant nodules that had neither doubled nor progressed in a given month were at risk of doubling or doubling/progressing (i.e., from local to regional, from regional to distant) in a subsequent month. Based on the calculated values (and assuming no diagnostic intervention), mean tumor doubling time was estimated to be 5.3 months in base-case analyses, while 55.3% of patients were estimated to experience disease progression during the two-year follow-up period.

Although the Gould calculations were based on volume doubling time data from a study published in 1973, they were deemed suitable for use in both the Gould cost-effectiveness analysis and a more recent cost-effectiveness analysis by Deppen and colleagues [20–22]. Moreover, the mean tumor volume doubling time in the Steele study—157 days—is only modestly greater than the mean VDT of lung cancers detected in clinical practice and those detected by chest x-ray screening, as reported in a review—136 days and 150 days, respectively [23]. Additional details regarding the derivation of tumor growth/progression may be found in the S1 Appendix.

### Overdiagnosis

A certain proportion of incidentally detected lung cancers will be indolent in nature, i.e., so slowly progressing, that they would otherwise never come to clinical detection. If detected, indolent cancers typically have a highly favorable prognosis and would not result in death. For this reason, the early diagnosis and treatment of indolent lung cancer was assumed to confer no life-expectancy benefit to the patient while generating the same costs as the diagnosis and treatment of aggressive tumors. Because the extent to which this phenomenon would occur with and without use of AABT in clinical practice is unknown, we assumed that 18% all incidentally detected malignant nodules were indolent based on data from the National Lung Screening Trial (NLST) [24]. We believe this assumption to be conservative since overdiagnosis bias may be lower with incidentally detected nodules than with screen-detected nodules. Furthermore, since all lung cancers are assumed to be diagnosed in the model regardless of the strategy being analyzed, overdiagnosis occurs equally frequently in both. Thus, its only effect is to decrease the effective prevalence (risk) of aggressive lung cancer.

### Life expectancy (Unadjusted and quality-adjusted)

Life expectancy for patients with lung cancer was based on age, disease stage, and estimated risks of death from lung cancer and all other causes. Risk of death from lung cancer was estimated by combining data from The Surveillance, Epidemiology, and End Results (SEER) Program of the National Cancer Institute and data on relative survival from the National Cancer Database [25–26]. Because risk of progression in our analysis was based on a three-stage model of lung cancer (local, regional, and distant), while our life-expectancy estimates were based on the standard four-stage model, we assumed that the proportions of regional lung cancer cases that were stage 2 and stage 3 were similar to the ratio of these stages in clinical practice [25]. To the risk of death from lung cancer was added the risk of death from all other causes, taking into account the percentage of smokers in the model population and the increased risk of death from causes other than lung cancer among smokers [27–29]. Moreover, in the AABT strategy, additional patients are false-positive and undergo unnecessary biopsy with a small (1.1%) risk of death, which was incorporated into analyses [30].

Life expectancy for patients without lung cancer was based on data from the National Lung Screening Trial (NLST) [31]. Life-expectancies from NLST were not used for persons with lung cancer because they were substantially greater than those reported for US clinical practice. Investigators speculate that these improved life expectancies might have been due to the healthy volunteer effect (persons participating in a clinical trial are healthier than otherwise similar non-participants), stage migration (within a given stage, cancers are detected earlier by screening than in practice, e.g., stage I cancers are significantly smaller on average), and possibly better treatment at the academic centers participating in NLST.

Quality-adjusted life expectancy for patients with lung cancer were calculated by combining estimates of life expectancy and stage-specific utility values, which were based on data from the NLST [31]. For patients without lung cancer, quality-adjusted life expectancy was calculated by combining estimates of life expectancy and age-specific utility values from the published literature [32–33].

### Healthcare costs

Healthcare costs (2016 US$) included those for the evaluation of incidentally detected nodules (AABT, $575; periodic CT, $245), diagnostic follow-up ($5,415), and cancer treatment ($36,724) [31, 34–38]. Estimates for all of these parameters were either taken directly from published studies or derived based on data reported in published literature.

Absent data on the frequency with which the different biopsy procedures are performed in clinical practice, we assumed that bronchoscopic biopsy would be uncommonly used and that newer techniques for guided bronchoscopy—which have greater diagnostic yield but are currently limited to relative few specialized centers—would not be performed. We further assumed that CT-guided trans-thoracic needle biopsy would be the most common biopsy technique.

Our estimated cost of lung cancer treatment was derived using published data from the NLST, updated to 2016 costs [31]. In the NLST analysis, treatment costs were based on utilization specific to cancer treatment for 5,133 trial patients over the entire period of follow-up. Although we believe these NLST treatment cost estimates to be the best available, we note that costs were not specified by stage of lung cancer, perhaps because there was little difference in total costs of lung cancer by stage from diagnosis to end of follow-up or death, a period during which treatment costs of patients diagnosed in early stages who later progress are captured. Evidence for this lack of difference is the fact that mean costs were similar in the CT-screening and x-ray-screening arms, despite substantial differences in the distribution of lung cancers by stage in the two arms. Since other published data on lung cancer treatment costs indicate substantial differences by stage at diagnosis, we examined the impact of varying treatment costs and the ratio of early-stage to late-stage treatment costs in sensitivity analyses. Further discussion of treatment costs may be found in the S1 Appendix.

### Analyses

Life expectancy (unadjusted and quality-adjusted) and expected costs were calculated for 1,000 persons in each of the two strategies. Cost-effectiveness was calculated using the ratio of the difference in expected costs to the corresponding differences in life-years and quality-adjusted life-years for the AABT strategy and no-AABT strategy, respectively. The perspective of the analysis was the US healthcare system; future costs and life-years were discounted at 3% per year.

The robustness of model projections to changes in several key assumptions and parameter estimates were examined in sensitivity analyses. Lung cancer prevalence, initial distribution of malignant nodules, AABT cost, AAB sensitivity/specificity, tumor doubling time, disease progression, overdiagnosis bias, follow-up cost, and treatment cost were all varied across reasonable alternative values. In addition, the robustness of results were evaluated when using an alternative schedule for CT screening, namely that recommended in the Fleischner Society guidelines (i.e., CT at 3, 9, and 24 months, respectively) [2].

## Results

### Base-case analyses

Among 1,000 patients with incidentally detected nodules 8–30 mm, with an intermediate-risk of lung cancer, and evaluated by CT surveillance alone 95 (9.5%) were assumed to have lung cancer. With no use of the AABT (i.e., CT surveillance alone), 73.6% of patients with lung cancer would be diagnosed with local disease, 22.0% with regional disease and 4.4% with distant disease. Healthcare costs among all patients in the model population (n = 1,000) would total $4.0 million ($4,040 per person), and life years (discounted), 12,130 (12.13 per person) (Table 3).

**Table 3:** Outcomes (discounted) with use of AABT versus CT surveillance alone for early diagnosis of lung cancer in patients who have incidentally detected pulmonary nodules, are at intermediate risk, and were scheduled for CT surveillance alone*.

	CT Surveillance	AABT	Difference
Sensitivity/Specificity AABT = 41% / 93%			
Life-Years	12,130	12,183	53
QALYs	9,793	9,832	39
Total Cost	$4,039,582	$4,989,024	$949,442
Cost per Life-Year Gained	—	—	$18,029
Cost per QALY Gained	—	—	$24,330
Sensitivity/Specificity AABT = 28% / 98%			
Life-Years	12,130	12,167	37
QALYs	9,793	9,821	27
Total Cost	$4,039,582	$4,722,069	$682,487
Cost per Life-Year Gained	—	—	$18,454
Cost per QALY Gained	—	—	$24,833

AABT, autoantibody test; CT: computed tomography; QALY, quality-adjusted life-year

*Model population assumed to comprise 1,000 patients

With use of the AABT set at sensitivity/specificity of 41%/93% and, if negative, CT surveillance, there would be among the 1,000 patients: 842 true-negatives, 63 false-positives, 39 true-positives, and 56 false-negatives. Accordingly, immediate biopsy of the nodules with a positive AABT would result in a stage shift of 10.8% in comparison with the no-AABT strategy (i.e., there would be 10.8% fewer patients with regional/distant lung cancer in the AABT strategy: local, 84.4%; regional, 13.0%; distant, 2.6%). On the other hand, 63 patients would have a false-positive AABT and would unnecessarily incur the costs (and risks) of biopsy. On balance, therefore, expected costs would be higher by $949,442 ($949 per person) than in the no-AABT strategy, but life years would be higher by 53 (0.053 per person), resulting in a cost per life-year gained of $18,029 and cost per QALY gained of $24,330.

With use of the AABT set at a sensitivity/specificity of 28%/98% (and, if negative, CT surveillance), there would be: 887 true-negatives, 18 false-positives, 27 true-positives, and 68 false-negatives, and the corresponding stage shift, 7.4% (i.e., there would be 7.4% fewer patients with regional/distant lung cancer in the AABT strategy: local, 81.0%; regional, 15.9%; distant, 3.2%). Compared with the no-AABT strategy, expected costs would be higher by $682,487 ($682 per person) and life years would be higher by 37 (0.037 per person), resulting in cost-effectiveness ratios of $18,454 per life-year gained and $24,833 per QALY gained.

### Sensitivity/scenario analyses

Table 4 reports cost-effectiveness ratios (i.e., cost per QALY gained) when using various alternative values for the aforementioned model parameters. The cost-effectiveness of using AABT for incidentally detected pulmonary nodules in this intermediate-risk CT-surveillance population was sensitive to the prevalence of malignancy, the sensitivity/specificity of the AABT, and the probability of stage progression among malignant nodules. The impact of changes in AABT sensitivity on cost-effectiveness is mediated—to a large extent—via the corresponding stage shift. For example, when increasing AABT sensitivity from 41% to 50% (holding specificity at 93%), the number of true-positives increases (and thus the number of false-negatives decreases) by 9 and the resulting stage shift increases from 10.8% to 13.2%; the cost per QALY gained decreases from $24,330 to $19,743. The cost-effectiveness of AABT was insensitive to all cost parameters, except for the cost of the test itself. Variation in the degree of over-diagnosis bias had little effect on cost-effectiveness ratios, and cost-effectiveness ratios were somewhat lower when using the CT screening schedule recommended in the Fleischner guidelines.

**Table 4:** Sensitivity analyses on cost-effectiveness (cost per QALY gained) of using AABT versus CT surveillance alone for early diagnosis of lung cancer in patients who have incidentally detected pulmonary nodules, are at intermediate risk, and were scheduled for CT surveillance alone.

	Sensitivity / Specificity of AABT	
	41% / 93%	28% / 98%
Basecase	$24,330	$24,833
Prevalence of Lung Cancer (basecase = 9.5%)		
3.0%	$90,973	$83,880
12.0%	$18,821	$19,479
Distribution of Malignant Nodules at Detection (basecase = 100% local)		
87.5% local / 12.5% regional	$27,994	$28,444
Cost of AABT (basecase = $575)		
$124	$12,773	$8,423
$325	$17,923	$15,737
$450	$21,127	$20,285
$750	$28,814	$31,201
Cost of CT (basecase = $245)		
$500	$24,989	$25,019
$1,500	$27,575	$25,749
Sensitivity—AABT (basecase = 41% / 28%)		
20%	$52,956	$35,078
30%	$33,952	$23,143
40%	$24,974	$17,266
50%	$19,743	$13,768
Specificity—AABT (basecase = 93% / 98%)		
90%	$29,107	$44,300
100%	$13,994	$20,485
Tumor Doubling Time (basecase = 5.3 months)		
3.8 months	$18,663	$19,265
7.4 months	$31,360	$31,560
Probability of Disease Progression* (basecase = 55.3%)		
33.6%	$61,501	$59,997
79.1%	$12,331	$12,777
Overdiagnosis Bias (basecase = 18%)		
13.0%	$22,875	$23,391
23.0%	$25,982	$26,476
Cost of Diagnostic Follow-Up (basecase = $5,415)		
$4,061	$22,132	$23,942
$6,769	$26,528	$25,725
Cost of Lung Cancer Treatment (basecase = $36,724)		
$27,543	$24,287	$24,792
$45,905	$24,373	$24,875
Stage 1 = $27,543 / Stage 4 = $45,905	$23,677	$24,266
CT Screening Schedule (basecase = 4, 10, 21 months)		
3, 9, 24 months	$21,224	$21,649

AABT, autoantibody test; CT, computed tomography; NSCLC, non-small cell lung cancer; SCLC, small cell lung cancer

*During 2-year follow-up

## Discussion

The evaluation of incidentally discovered pulmonary nodules is an increasingly common problem for clinicians. For nodules with diameter 8–30 mm and an estimated intermediate-risk of malignancy, the optimal strategy for evaluation is especially problematic. Several studies have indicated that published guidelines for nodule evaluation are not followed in a substantial proportion of cases. In particular, the recommendation from the ACCP for PET-CT evaluation of such nodules is often ignored in favor of CT surveillance alone. In the introduction, we noted concerns about the accuracy of PET-CT as one of the major reasons for failure to follow guidelines. Iaccarino and Wiener have discussed additional possible reasons for this pattern, including ignorance of guidelines, disagreement with guidelines due to their relatively weak evidentiary basis, patient characteristics (e.g., severe chronic obstructive pulmonary disease) and preferences, and diagnostic resource availability [5]. In this study, we analyzed the cost-effectiveness of the AABT as an aid to diagnosis among patients in this risk group, as compared with CT surveillance. Study results indicate that use of the AABT results in earlier diagnosis for a small proportion of patients with malignant nodules and a consequent beneficial stage shift. This benefit is associated with some extra costs, however, namely the costs of AABT testing and the unnecessary biopsies of the relatively small number of persons with benign nodules whose AABT tests are positive (false-positives).

On balance, the results of this study indicate that a strategy of administering this test to all such patients would be cost-effective by conventional criteria in comparison with CT surveillance alone. In our base-case analyses of intermediate-risk patients scheduled for CT surveillance with a 9.5% prevalence of malignancy, the cost-effectiveness of the AABT strategy was $24,330 to $24,833 per QALY gained, depending on which version of the test was used. In groups of patients with higher prevalence of lung cancer, cost-effectiveness ratios are even more favorable. Cost-effectiveness ratios less than $50,000 per QALY have long been considered to be a worthwhile investment of scarce healthcare resources in the US, and much higher thresholds for cost-effectiveness in the current era have been advocated ($100,000-$150,000) [39]. We note that our estimates of cost-effectiveness may actually be somewhat conservative, because the pulmonologists and thoracic surgeons who selected patients for CT surveillance in the Tanner study were participating in a clinical trial of a blood biomarker and may have had greater expertise in risk assessment and nodule management than would a random sample of physicians managing such nodules. Their expertise may have resulted in a lower prevalence of lung cancer among patients scheduled for CT surveillance alone, and thus a higher cost-effectiveness ratio, than would be the case in typical clinical practice.

As is true of all such models, ours is based on a number of assumptions, the validity of which is uncertain. Probably the greatest uncertainty concerns the risk of progression. Data concerning the natural history of early-stage lung cancer are very limited, and a survey of experts revealed considerable variation in beliefs about lung cancer growth and progression [40]. However, support for the substantial risk of progression used in our analysis comes from a report of survival among patients with stage 1 non-small cell lung cancer (NSCLC) [41]. Among patients for whom surgical treatment was recommended but declined, median relative survival was 20 months, and among all untreated patients, those whose tumors measured < 2 cm in diameter survived only modestly longer than those with larger tumors—15 months versus 12 months. There is also uncertainty concerning some of the other parameter estimates, especially the cost of diagnostic evaluation (e.g., the frequency with which the various types of biopsy are employed in clinical practice) and the cost of lung cancer treatment (e.g., variation across stages). We note, however, that—aside from discounting—these costs are largely the same in each of the alternative strategies considered and sensitivity analyses revealed that cost-effectiveness ratios are insensitive to these parameters.

In summary, this study suggests that using the AABT as an aid in the early diagnosis of lung cancer in patients with incidentally detected solid pulmonary nodules 8–30 mm and an intermediate-risk of malignancy is likely to be cost-effective by current standards in comparison with the common current practice of CT surveillance alone for the initial evaluation of such nodules.

## Supporting information

S1 Appendix Cost-effectiveness of an autoantibody test (*Early*CDT-Lung) as an aid to early diagnosis of lung cancer in patients with incidentally detected pulmonary nodules. (DOC)
