# Supplementary material for: Cost-effectiveness of an autoantibody test (EarlyCDT-Lung) as an aid to early diagnosis of lung cancer in patients with incidentally detected pulmonary nodules
Source: PLoS One. 2018 May 22;13(5):e0197826. doi: 10.1371/journal.pone.0197826 (PMC5963796; doi:10.1371/journal.pone.0197826)
Supplement: S1 Appendix — (DOC) [file pone.0197826.s001.doc]

**S1 Appendix:**

**Cost-Effectiveness of an Autoantibody Test (*Early*CDT-Lung) as an Aid to Early Diagnosis of Lung Cancer in Patients with Incidentally Detected Pulmonary Nodules**

**Likelihood Ratio of a Positive Test.** The likelihood ratio of a positive test is calculated as the sensitivity of the test divided by 1 minus the specificity. For the AABT with a sensitivity of 28% and a specificity of 98%, the likelihood ratio associated with a positive test is 14. To determine the increase in the probability that a nodule is malignant, the probability of the nodule being malignant prior to the test, i.e. the estimated risk (converted to odds for mathematical purposes,) is multiplied by the likelihood ratio to obtain the odds of the nodule being malignant in the presence of a positive test (the posterior odds) [1]. For example, if the probability of malignancy was estimated to be 12.5% prior to the test (odds of 1/7), then a positive test would alter the odds to 14/7 (probability of 67%).

**Prevalence of Lung Cancer among Intermediate-Risk Patients Scheduled for CT Surveillance.** Table 1 in the Tanner study indicates that there were 12 patients with cancer among all patients who were scheduled for CT surveillance (all three risk subgroups combined). Table 2 and Table 3 report study data by estimated risk subgroup. Table 2 includes data for the low-risk and high-risk subgroups only, and indicates that no patients at low risk and 5 patients at high risk had lung cancer and were assigned to CT surveillance. Thus, there must have been 7 patients with lung cancer in the intermediate-risk subgroup assigned to CT surveillance (12 minus 5). Table 3 indicates that 74 of the 174 patients in the intermediate-risk subgroup were scheduled for CT surveillance, yielding a lung cancer prevalence of 9.5% (7/74).

**Growth/Progression of Malignant Nodules.** The probability of a given nodule doubling by each month of the modeling horizon was estimated using data from Gould 2003 (appendix Figure 5 in original article [below]) [2]. Using the same figure, the probability of progressing—given the tumor has doubled—by month was estimated by fitting a Poisson regression and extrapolating at given monthly intervals and calculating the difference from month N to month N+1. This difference was the estimated probability of progressing.


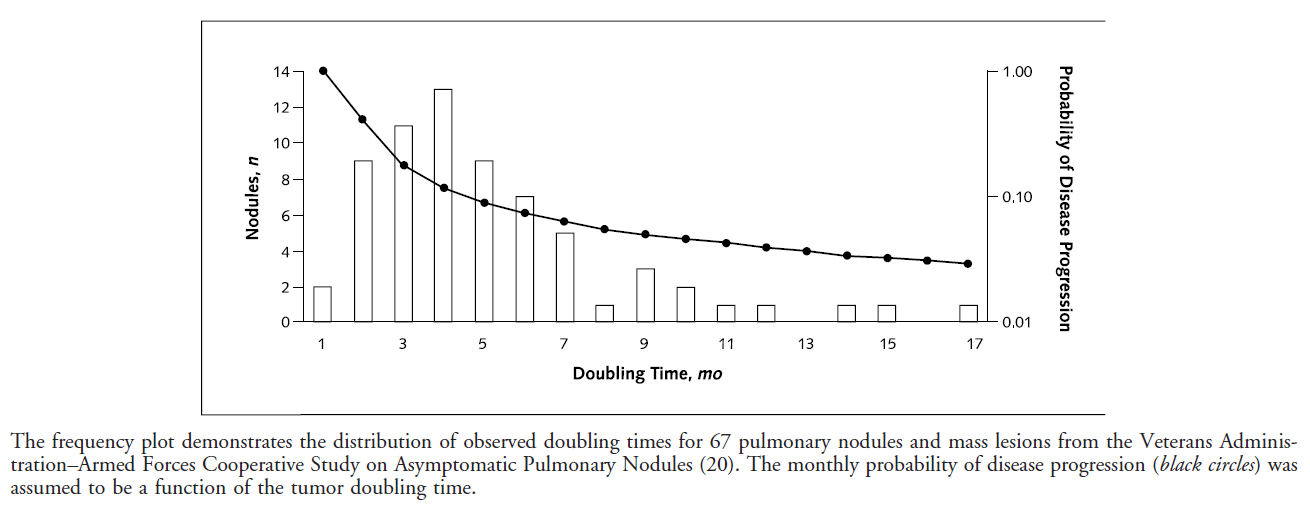


The probabilities were then combined with sensitivity estimates for AABT to calculate the expected distribution of nodules after 24 months (see figure below). At the time of the initial AABT, the sensitivity estimate would “siphon” off those persons with malignant nodules and positive test results and they would be assumed to be diagnosed and treated. Those not detected were subjected to the monthly probabilities estimated as described above and would undergo periodic CT scans. Tumors that doubled were assumed to be detected by the CT scan and siphoned from the population. Those tumors that were siphoned were assumed to have no further chance of progression. The number of tumors by stage was then tallied at the end of 24 months to calculate the expected stage distribution of the strategy. Differences between strategies were calculated to be the estimated stage shift.

**Healthcare Costs.** Costs included those for diagnostic tests: AABT, periodic CT, biopsy, and treatment of lung cancer (Table 2). While the current costs of CT, AABT, and biopsy could be established with some certainty from published sources, as could the performance characteristics of the different biopsy techniques for nodules <20 mm in diameter, the cost of biopsy could vary substantially in the model depending on the frequency with which the different biopsy procedures are performed. Absent data on such frequencies, we assumed that bronchoscopic biopsy would be infrequent and that newer techniques for guided bronchoscopy, which have greater diagnostic yield but are currently limited to relative few specialized centers, would not be performed. We assumed that CT-guided trans-thoracic needle biopsy would be the most common biopsy technique. We point out that total biopsy costs for lung cancers are the same in both scenarios and that the only difference in biopsy costs between the scenarios arises from the relatively small number of biopsies attributable to AABT- false–positives in the AABT scenario. The effect of biopsy costs on cost-effectiveness was analyzed by varying the expected cost of biopsy in sensitivity analysis.

The cost of lung cancer treatment was taken from the NLST cost-effectiveness study, updated to 2016 US dollars [3]. In the NLST analysis, treatment costs were not specified by stage. The lack of data by stage may reflect the fact that, since medical costs were tallied from diagnosis until the end of follow-up, and thus the terminal costs of patients who were diagnosed in early stages and later progressed were captured, there was little difference in total costs by stage at diagnosis. Evidence for this lack of difference is the similar average per patient costs in the CT-screening and x-ray-screening arms, despite substantial differences in the distribution of lung cancers by stage in the two arms. Whether or not lung cancer treatment costs from diagnosis to death vary by stage at diagnosis is unclear, however. Recent cost data from the US by stage is limited to monthly costs by phase of care-- initial, continuing, terminal; these data suggest higher costs for advanced stages [4]. Recent data for the first two years of treatment in Canada and the first three years of treatment in Spain indicate that treatment of advanced cancer is more expensive in the short-run [4-6]. We examined the impact of varying mean treatment cost and the ratio of early-stage to late-stage treatment costs in sensitivity analyses.

**References**

1. McGee S. Simplifying likelihood ratios. J Gen Intern Med. 2002;17: 647-50.
2. Gould MK, Sanders GD, Barnett PG. Cost-effectiveness of alternative management strategies for patients with solitary pulmonary nodules. Ann Intern Med*.* 2003;138: 724-35.
3. Black WC, Gareen IF, Soneji SS, Sicks JD, Keeler EB, Aberle DR, et al. Cost-effectiveness of CT screening in the National Lung Screening Trial. N Engl J Med*.* 2014;371(19): 1793-802.
4. Cipriano LE, Romanus D, Earle CC. Lung cancer treatment costs, including patient responsibility, by disease stage and treatment modality, 1992 to 2003. Value Health. 2011;14(1): 41-52.
5. Cressman S, Lam S, Tammemagi MC. Resource utilization and costs during the initial years of lung cancer screening with computed tomography in Canada. J Thorac Oncol. 2014;9(10): 1449-58.
6. Corral J, Espinàs JA, Cots F. Estimation of lung cancer diagnosis and treatment costs based on a patient-level analysis in Catalonia (Spain). BMC Health Serv Res. 2015;15: 70.
